# Adult Prader-Willi Syndrome: An Update on Management

**DOI:** 10.1155/2016/5251912

**Published:** 2016-06-08

**Authors:** Luk Ho-Ming

**Affiliations:** Clinical Genetic Service, Department of Health, 3/F Cheung Sha Wan Jockey Club Clinic, 2 Kwong Lee Road, Shamshuipo, Kowloon, Hong Kong

## Abstract

With the advancement of medical care, the survival of most patients with syndromal genetic disease is greatly improved. In this case report, we have reported an adult Prader-Willi syndrome patient who is being diagnosed at the age of 33. The clinical features and their associated complications during adulthood have been reviewed.

## 1. Introduction

Prader-Willi syndrome (PWS) is a recognizable syndromic form of neurodevelopmental disorder. It is the most common congenital imprinting disease. The incidence is about 1 in 15,000–25,000 live births [1]. Although most of them are diagnosed during childhood, with the improvement of medical care, it is not uncommon for adult physicians to encounter PWS patients. Apart from obesity-related complications, behavioral problems and psychiatric manifestations are common among them. Because of these, a syndrome specific surveillance and management are recommended.

## 2. Case Presentation

A 33-year-old gentleman was referred from adult psychiatrist to genetic clinic for assessment of his intellectual disability. He was the first child of a nonconsanguineous Chinese couple, born at full term with birth weight of 3 kg (10–25th percentile) through normal vaginal delivery in rural hospital of China. His neonatal course was complicated with poor feeding and bilateral undescended testis. His failure to thrive and hypotonia were gradually improved during early infancy. The growth parameters and appetite were increased significantly in early childhood that he became obese afterwards. He had developmental delay and studied in special school till the age of 12. He came to Hong Kong at the age of 20 and developed type 2 diabetes mellitus since he was 22 years old. He was regularly followed up in endocrine clinic for his diabetes and obesity. Endocrine investigation showed borderline low testosterone level of 200 ng/dL (normal 240–950 ng/dL) with HbA1c of 9.4% (normal < 6%). His intellectual function assessed by Wechsler Adult Intelligence Scale-Fourth Edition (Hong Kong) showed he had severe grade mental retardation. He also had obsessive compulsive behavior that required psychotherapy.

**Figure 1:**
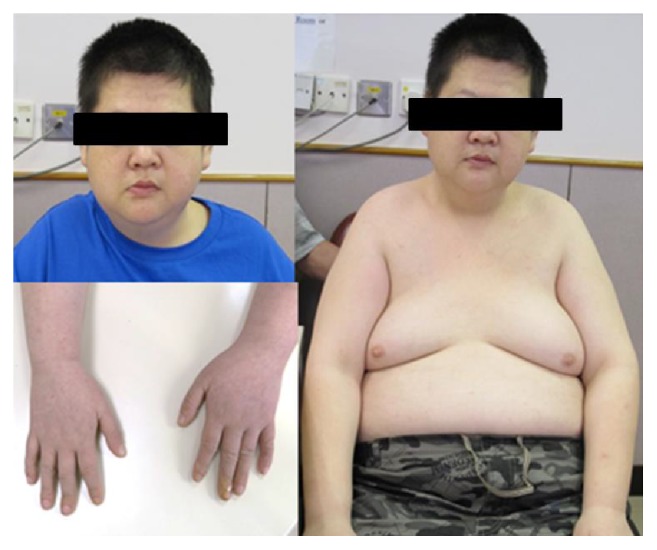
The clinical photos showed there is no facial dysmorphism in this Chinese gentleman, but with fair complexion, bilateral gynecomastia, truncal obesity, and relative small hands.

Clinical assessment in genetic clinic showed he was obese with body weight of 76.8 kg (90–97th percentile), body height of 152 cm (6 cm < 3th percentile), and the BMI of 33.2 Kg/m^2^. There were no facial dysmorphic features except that he had fair complexion. He had bilateral gynecomastia. Both hands and feet were relative small (Figure 1). There was only scanty axillary and pubic hair. Stretched penile length was 4 cm and two 5 mL testes were palpable. There were no acanthosis nigricans or other evidence of insulin resistance. The rest of the physical examination was essentially normal. Based on the medical history and physical findings, Prader-Willi syndrome (PWS) was suspected. Methylation study using SALSA MLPA ME028 Prader-Willi/Angelman probemix kit from MRC-Holland (Amsterdam, Netherlands) was performed. It showed heterozygous deletion and hypermethylation of* SNRPN* locus. The 15q11–13 deletion was confirmed by FISH study; thus the diagnosis of Prader-Willi syndrome due to paternal deletion of* SNRPN* locus was substantiated. He was then referred to endocrine clinic for assessment of growth hormone usage and testosterone replacement.

## 3. Discussion

Prader-Willi syndrome (PWS) is a complex neurodevelopmental disease that is caused by absence of paternal expressed imprinting genes at chromosome 15q11–13 region. The typical clinical features included hypotonia, poor feeding, and hypogonadism during neonatal period; characteristic facial gestalt, short stature, small hands and feet, and hyperphagia during early childhood; and obesity, learning, and behavior problems at their later life. Although this is usually diagnosed in paediatric population as recognizable syndrome, some of them would attend to medical care during adulthood. With the improvement of medical care, the life expectancy of patients with PWS has improved significantly. Therefore, it is not unusual for medical practitioner to encounter adult PWS during their clinical practice.

The diagnosis of adult PWS is challenging. Some of those characteristic clinical features like almond shapes eyes, small hands and feet, hypogenitalism, and skin picking behavior may disappear with age. Majority of adult PWS patients had intellectual disability that the made clinical history was not reliable. As a result, many adult PWS are being undiagnosed or misdiagnosed as other genetic diseases [2].

A high index of suspicion for PWS is necessary if the adult patient presented with the signs and symptoms in Table 1 [3].

The mortality rate of adult PWS is approximately 3% per year [4] and the average age of death was 33 years [5]. Early diagnosis of adult PWS is important, as the specific features of adult PWS like high pain threshold, abnormal thermoregulation, and paucity of vomiting need special care, particular during acute illness [2, 6]. Although the use of growth hormone would improve the body composition, quality of life, and cognitive function in adult PWS patients, it is not without side effects. Therefore, anticipatory guidance and surveillance for adult PWS are necessary. The common medical problems in adult PWS are summarized in Table 1 [3].

With more vigilance in clinical suspicion and the usage of genetic test, together with better understanding of their natural history and age related complications, a PWS specific surveillance and management as suggested would lead to a better quality of medical care in adult PWS patients.

## Figures and Tables

**Table 1:** Clinical features and common medical problems in adult PWS [3].

Signs and symptoms that warrant referral to genetic service for genetic assessment of PWS in adult	
History of hypotonia, poor sucking, and feeding problems during infancy	
Short stature	
Hypogenitalism	
Hyperphagia	
Obesity	
Small hands and feet	
Intellectual disability and/or behavioral problems	
Thick, viscous saliva	

Common medical problems in adult PWS	
System	Medical problems

Cardiovascular	Hypertension
	Hyperlipidemia

Respiratory	Breathing related sleep disorder
	Infection like pneumonia

Endocrine	Type 2 diabetes mellitus
	Hypothyroidism
	Hypogonadism

Psychological	Psychosis
	Behavioral problems
	Sexuality
	Abuse

Dermatological	Skin picking
	Soft tissue infection like erysipelas

Musculoskeletal	Osteoporosis

Iatrogenic	Growth hormone related side effects
